# Phosphorus ingestion improves oral glucose tolerance of healthy male subjects: a crossover experiment

**DOI:** 10.1186/s12937-015-0101-5

**Published:** 2015-10-29

**Authors:** May Khattab, Christelle Abi-Rashed, Hala Ghattas, Sani Hlais, Omar Obeid

**Affiliations:** 1 Department of Nutrition and Food Sciences, American University of Beirut, Beirut, Lebanon; 2 Department of Family Medicine, American University of Beirut, Beirut, Lebanon

**Keywords:** Phosphorus, Glucose, Area under the curve, OGTT, Insulin sensitivity

## Abstract

**Background:**

Fasting serum phosphorus (P) was reported to be inversely related to serum glucose and insulin, while the impact of P ingestion is not well documented. The effect of P intake with or before glucose ingestion on postprandial glucose and insulin statuses was investigated.

**Method:**

Two cross over experiments using healthy male subjects were conducted. *Experiment 1*: Overnight fasted subjects (*n* = 7) randomly received: 500 mg of P tablets, glucose (75 g) solution with placebo or 500 mg of P tablets. *Experiment 2*: Overnight fasted subjects (*n* = 8) underwent similar procedures to those of experiment 1, except that placebo or 500 mg P tablets were given 60 min prior to glucose ingestion.

**Results:**

In both experiments, serum P decreased following glucose ingestion. Co-ingestion of P with glucose improved, at time 60 min, postprandial glucose (*P* < 0.05), insulin (*P* < 0.05), and insulin sensitivity index (*p* < 0.006), while P pre-ingestion failed to exert similar effect.

**Conclusion:**

This study suggests that postprandial glucose and insulin are affected by exogenous P supply, especially when co-ingested with glucose.

## Introduction

Over the past few decades, the high prevalence of metabolic syndrome has become a worldwide phenomenon, associated with an increased risk and prevalence of type 2 diabetes mellitus [1]. This increasing phenomenon has been found to be associated with dietary and lifestyle changes, which favor the consumption of phosphorus (P)-deficient food items [2]. In parallel, plasma phosphate levels were found to be synergistically related to glucose tolerance and insulin sensitivity [3–5]. The association between P and glucose is believed to be mediated by insulin [6], as a result of its capacity to stimulate both P uptake by the muscles [7] and phosphorylation of many compounds; thereby, creating a competition [8] among these compounds for P.

Additionally, overweight and/or obesity, a known risk factor for type 2 diabetes mellitus, is reported to be inversely related to plasma P [2, 3]. The intakes of high protein food choices, which are rich sources of P, have been reported to improve weight loss [9] by a calcium independent mechanism [10, 11]. Furthermore, the intake of dairy products has been also found to be associated with a lower risk for type 2 diabetes mellitus in three meta-analyses of large prospective epidemiological studies [12–15]. Moreover, since post-prandial glycaemia is a risk factor for type 2 diabetes mellitus [16, 17], it is reasonable to postulate that P is involved in its development. Several dietary, lifestyle and pharmaceutical approaches have been used to reduce postprandial glycaemia, including the enhancement of intracellular glucose trapping. Recently glucokinase activators have been developed and tested, but their capacity to improve glycaemia was associated with several side effects [18–20] that may be attributed to hepatic intracellular P depletion, especially since a limited quantity of free P is present intracellularly.

In brief, increased incidence of glucose intolerance and type 2 diabetes mellitus is associated with dietary changes that favor a reduction in the consumption of P, an important mediator in glucose metabolism [5, 21–23]. Despite the presence of evidence showing the inefficiency of fasting P to reflect its diurnal [24] or postprandial status [25], a positive association between fasting P levels and insulin sensitivity has been reported [26].

Micronutrients enrichment of carbohydrate meals was reported to reduce post-prandial glycaemia [27]. Although P is tightly integrated in the metabolism of glucose, the impact of P ingestion on glucose tolerance and insulin resistance of healthy subjects is not well documented. However, phosphate infusion of healthy subjects was reported to improve insulin sensitivity under euglycaemic conditions [6] and phosphate supplementation of hypophosphatemic glucose intolerant patients improved glucose tolerance [28]. The aim of this study was to investigate the effect of exogenous P on postprandial glucose and insulin status in healthy subjects. Based on that, this study holds significance in the advances of human nutrition in improving glucose tolerance and therefore preventing the development of type 2 diabetes mellitus in humans.

## Subjects and methods

The study was comprised of two experiments. Healthy male subjects were recruited to perform the experiments (Table 1). Placebo (cellulose) or P (125 mg of potassium phosphate per tablet) tablets, which had similar weight and color, were administered to subjects in a randomized order to prevent order-of-treatment effect. The study protocol was approved by the Institutional Review Board committee at the American University of Beirut, in addition to consent forms that have been obtained from patients.

**Table 1:** Characteristics of the study subjects

Characteristics	Mean ± SEM
Experiment 1 (*n* = 7)	
Age (years)	23.22 ± 1.83
Weight (kg)	68.88 ± 4.05
Height (m)	1.74 ± 0.02
BMI (kg/m^2^)	22.65 ± 0.82
Fasting glucose (mg/dl)	86.38 ± 1.62
Fasting triglycerides (mg/dl)	90.63 ± 15.9
Fasting phosphorus (mg/dl)	4.06 ± 0.19
Experiment 2 (*n* = 8)	
Age (years)	27.3 ± 1.68
Weight (kg)	73 ± 4.78
Height (m)	1.76 ± 0.04
BMI (kg/m^2^)	23.5 ± 0.97
Fasting glucose (mg/dl)	88 ± 2.17
Fasting triglycerides (mg/dl)	119 ± 15.3
Fasting phosphorus (mg/dl)	3.75 ± 0.2

### Experiment 1: the effect of phosphorus ingestion on oral glucose tolerance test [OGTT]

Seven overnight fasted subjects (age (mean ± SEM): 23.2 ± 1.83 years; BMI: 22.65 ± 0.82 kg/m^2^) (Table 1: Experiment 1) were asked to attend 3 experimental sessions that were separated by a minimum of 3 days. Sessions included the consumption of either 500 mg of P (4 tablets), a glucose solution (75 g glucose) with 4 Placebo tablets, or a glucose solution (75 g glucose) with 500 mg P. All were ingested with 250 ml of cold water. Blood was withdrawn at baseline and monitored till 240 min (min) after consumption.

### Experiment 2: the effect of pre-phosphorus ingestion on OGTT

Based on data from experiment 1, peak serum P concentration (60 min) was associated with significant decrease in serum glucose and insulin levels. Thus, experiment 2 was designed to investigate whether P intake one hour prior to glucose ingestion would potentiate the effect of P on postprandial glucose and insulin levels. Eight overnight fasted subjects (age: 27.3 ± 1.68 years; BMI: 23.5 ± 0.97 kg/m^2^) (Table 1: Experiment 2) attended 2 experimental sessions that were separated by a minimum of 3 days. Subjects were given placebo or P (500 mg) tablets 60 min prior to glucose ingestion. Blood was drawn at baseline (−60 min) and monitored till 240 min relative to glucose ingestion.

### Laboratory analyses

Serum was separated from collected blood samples and stored at −80 °C for later analysis of glucose, total P, triglyceride and insulin. Glucose was measured from venous samples. Insulin sensitivity was estimated by the method of Caumo et al. [29] and expressed as (x 10^4^ dl.kg^−1^.min^−1^.μUml^−1^). The method is based on the kinetic of both glucose and insulin through coupling their rate of appearance into circulation following oral glucose ingestion. It is based on simple area under the curve type of calculation and was validated in normal subjects in whom their calculated insulin sensitivity was strongly correlated to that of frequentl formula y sampled iv glucose test (FSIGT) [29]. In addition, index of insulin sensitivity was calculated using the composite equation proposed by Matsuda and DeFronzo [30].

### Statistical analyses

Data are presented as means ± SEM. Paired t-tests were used to compare between treatments and to detect the difference from baseline within each treatment. Repeated Measure ANOVA was run to test the effect of treatment groups over time on each of the dependent variables (P, Glucose and insulin). The level of significance was fixed at *P* < 0.05.

## Results

Baseline serum levels of the different parameters (total P, glucose, insulin) were similar between sessions and both experiments (Table 1).

### Experiment 1

**Fig. 1:**
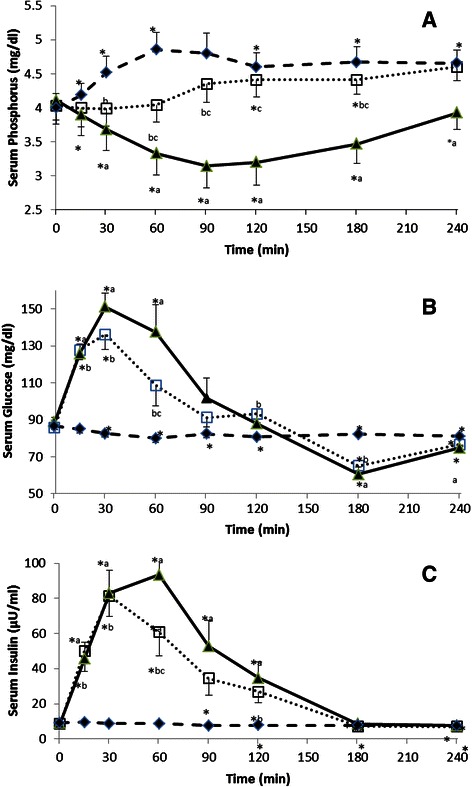
Changes in Serum Phosphorus (**a**), Glucose (**b**) and Insulin (**c**) levels of subjects in experiment1. #Experiment 1: After the ingestion of 500 mg phosphorus (-♦-), 75 g glucose (-▲-) or co-ingestion glucose + phosphorus (75 g glucose + 500 mg of phosphorus) (..□..). * *p-*value < 0.05: Paired t-test in the same treatment in comparison with baseline (time 0) value. ^a^ *p-*value < 0.05: Paired t-Test, phosphorus vs glucose treatments at each time point. ^b^ *p-*value < 0.05: Paired t-Test, phosphorus vs glucose + phosphorus treatments at each time point. ^c^ *p-*value < 0.05: Paired t-Test, glucose vs glucose + phosphorus treatments at each time point

Ingestion of P alone increased serum P significantly, while ingestion of glucose alone decreased postprandial serum P levels. The pattern of serum P changes following glucose and P ingestion (G + P) was different than that of the other two treatments (Fig. 1a), in line, repeated measures ANOVA showed that serum P was significant according to treatment (Table 2: Experiment 1). Postprandial serum glucose concentration of the P treatment group significantly decreased by around 5 mg/dl during the experimental session, but this minimal reduction is believed to be the result of fasting. Glucose ingestion increased postprandial serum glucose levels of both treatments, glucose and G + P, but the magnitude of the increase was significantly lower in the G + P as compared to glucose treatment at time 60 min (*P =* 0.016), (Fig. 1b). Repeated measures ANOVA showed that serum glucose levels were significantly different according to time, but failed to reach statistical significance between treatments (Table 2: Experiment 1).

**Table 2:** Repeated measure ANOVA outcome variables (Phosphorus, glucose and Insulin) of the two experiments

Outcome variables	Time	Treatment	Interaction
Experiment 1 (*n* = 7)^a^	(*P* value)	(*P* value)	(*P* value)
Serum Phosphorus	0.616	0.000	0.321
Serum Glucose	0.000	0.145	0.207
Serum Insulin	0.000	0.168	0.619
Experiment 2 (*n* = 8)			
Serum Phosphorus	0.001	0.002	0.498
Serum Glucose	0.000	0.815	0.961
Serum Insulin	0.000	0.233	0.724

^a^ Only glucose with placebo and glucose with P groups were included in the analysis

Ingestion of P alone did not alter postprandial insulin concentration. Insulin levels of the G + P treatment were significantly lower (*P =* 0.002) than that of the glucose treatment at time 60 min (Fig. 1c). Repeated measures ANOVA found that serum insulin was significant according to time only (Table 2: Experiment 1). Insulin sensitivity obtained from oral glucose tolerance test and according to Caumo et al. [29] formula increased following G + P treatment and the difference was close to significance (*P* = 0.051) (Table 3: Experiment 1). While index of insulin sensitivity increased significantly (*P* = 0.006) following the addition of P to OGTT (Table 3: Experiment 1). Insulin sensitivity of the P ingestion treatment was not determined since postprandial glucose and insulin levels were minimally affected.

**Table 3:** Measures of insulin sensitivity from oral glucose tolerance test

Outcome variables	Placebo	Phosphorus	Paired t-test (*P* value)
Experiment 1 (*n* = 7)			
Insulin sensitivity index [30]	5.69 ± 0.86	7.00 ± 1.06	0.006
Insulin sensitivity [29]	12.19 ± 3.85	20.22 ± 6.65	0.051
Experiment 2 (*n* = 8)			
Insulin sensitivity index [30]	9.17 ± 0.97	8.88 ± 0.78	0.633
Insulin sensitivity [29]	15.31 ± 2.58	18.39 ± 3.28	0.210

### Experiment 2

**Fig. 2:**
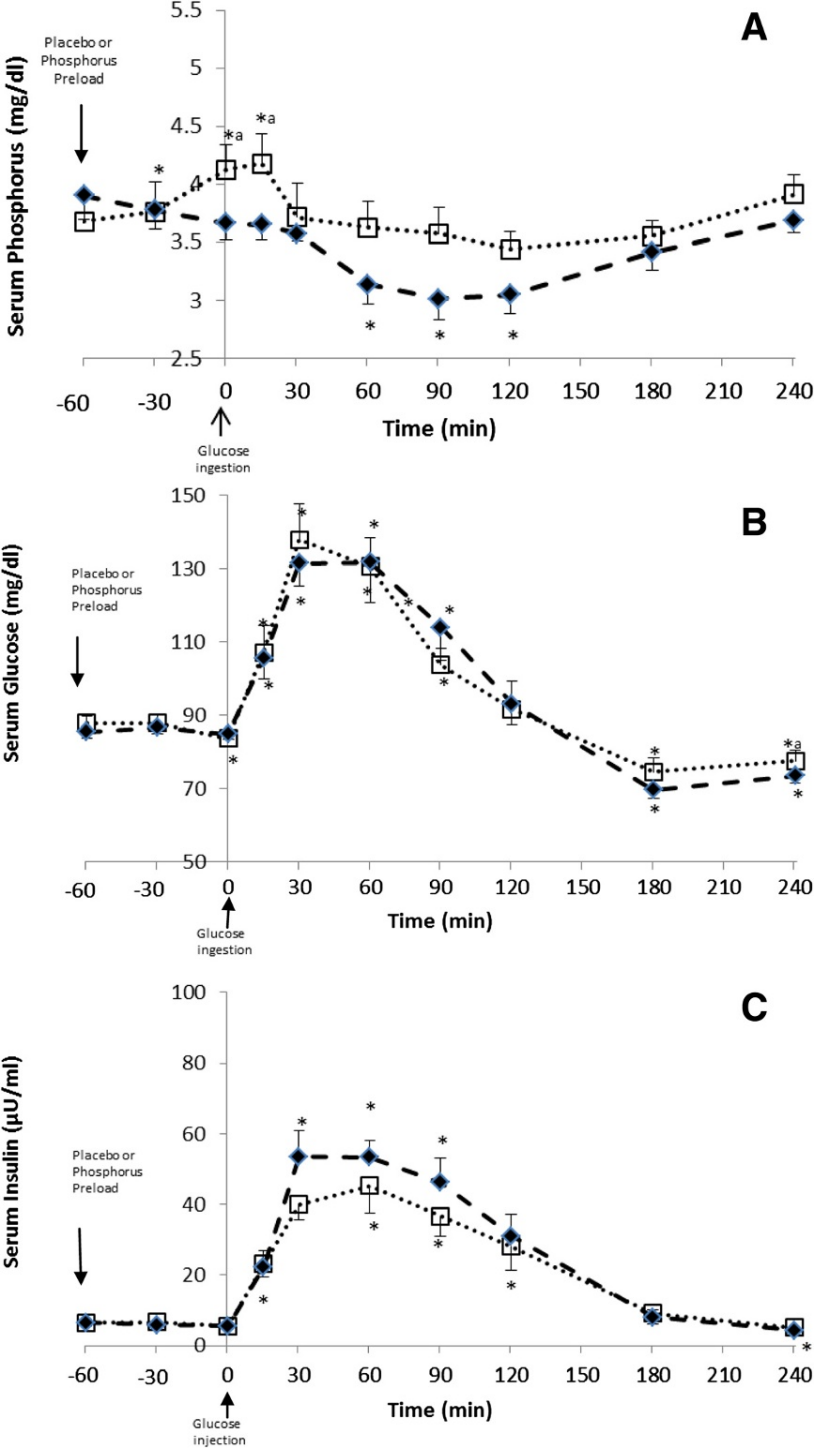
Changes in Serum Phosphorus (**a**), Glucose (**b**), and Insulin (**c**) levels of subjects in experiment2. #Experiment 2: After the he ingestion of 75 g glucose 60 min after placebo (-♦-) or 500 mg phosphors (..□..) preload. * *p-*value < 0.05: Paired t-test in the same treatment in comparison with baseline (time −60 min) value. ^a^ *p-*value < 0.05: Paired t-Test between placebo and phosphorus preload treatments at each time point

Ingestion of placebo tablets had no effect on serum P levels prior to glucose ingestion, but glucose ingestion decreased serum P levels (Fig. 2a). Following P ingestion, serum P levels increased significantly at time 0 and 15 min and then returned to baseline levels (Fig. 2a). Repeated measures ANOVA analysis of all time points showed that serum P was significant according to time and treatment (Table 2: Experiment 2). Serum glucose levels of the glucose and G + P treatments increased significantly following glucose ingestion (Fig. 2b). The increase in glucose levels was similar between the two treatments, except for a slight difference at time baseline and 240 min believed to be of no clinical significance (Fig. 2b). In line, repeated measures ANOVA were significant according to time only (Table 2: Experiment 2).

Glucose ingestion increased insulin levels of both treatments, but the magnitude of this increase was modestly lower in the P preload treated group (Fig. 2c) and repeated measures ANOVA failed to detect difference according to treatment (Table 2: Experiment 2). Measures of insulin sensitivity were found to be similar between the two treatments and this was expected since the changes in insulin and glucose were similar between the treatments (Table 2: Experiment 2).

## Discussion

In agreement with other studies, ingestion of P alone (experiment 1 and 2) increased postprandial serum P [31], while ingestion of grape juice [31], glucose alone [32–34], or other type of carbohydrate [35] reduced postprandial serum P levels. Glucose may indirectly affect P status through the stimulation of peripheral P uptake by insulin [7, 36, 37], which in turn stimulates the phosphorylation of several compounds including carbohydrate [35], fat and protein. About 60 % of infused P has been reported to be translocated from the extracellular to the intracellular compartment [6], mainly in skeletal muscles [21, 36]. This translocation is believed to be mediated via insulin action as glucose infusion in pancreatectomic dogs failed to induce a reduction in serum P except when insulin became available [21, 37, 38]. In line with this, the parallel increase in postprandial glucose and insulin (peaks at 30–60 min) was followed by a decrease in serum P (dip at 90 min). Therefore, under conditions of P intake alone, intracellular P is likely to have been affected since insulin was not altered.

In the current study, both co- and pre-ingestion of P were able to halt the drop in serum P levels following glucose ingestion. In the G + P treatment, increased P uptake and availability might have contributed to the drop in glucose and insulin levels at time 60 min, due to an increase in intracellular glucose trapping (phosphorylation), especially since insulin release depends on glucose circulation. This process might have played a role in the observed improvement in the measures of insulin sensitivity following P ingestion with glucose (Experiment 1). Phosphorylation or P trapping seems to have been substantially stimulated or became dependent on extracellular P 30 min after glucose ingestion; as indicated by the drop in postprandial serum P levels. This may partially be behind the failure of P pre-ingestion to impact postprandial plasma glucose, since the majority of phosphorus is known to be absorbed within 60 min, as supported by the finding from experiment 1. When glucose was ingested alone, the low availability of P may have hindered insulin phosphorylation capacity through creating competition for P. Such competition may have deleterious effects since it can affect glucose clearance and trapping, glycolysis and gluconeogenesis [39], and phospholipids and hepatic fat accumulation [40]. Therefore, postprandial glycemia and insulinemia seem to be improved by exogenous P availability and this may partially explain the reported association between low serum P with insulin resistance and elevated blood glucose levels [5, 23].

Ingestion of P before or with glucose was able to prevent the drop in postprandial serum P levels. The sustained high postprandial serum P in the G + P treatment in comparison to that of the glucose ingestion alone implies that intracellular P uptake may be controlled by a limited capacity for phosphorylation and/ or glucose uptake. In healthy subjects, peripheral glucose uptake, especially in skeletal muscles, is known to be triggered by insulin dependent Glut 4 stimulation [41]. While, intracellular glucose phosphorylation is controlled by the activities of glucokinase (liver) and hexokinase (muscle), the latter has a low Vmax (maximum velocity) capacity and is highly inhibited by glucose-P production [42]. The reduction in serum glucose of the G + P treatment argues against a defect in Glut 4 (glucose uptake), therefore the sustenance in plasma P may have been attributed to the low Vmax capacity of muscle hexokinase. Accordingly, ingestion of higher P doses would not be expected to further improve glucose, insulin, or insulin sensitivity.

The fact that P is absorbed along the entire intestinal tract [22, 43] could be responsible for the observed high plasma P levels (above baseline value) in the G + P treatment beyond the time (120 min) of availability of glucose and insulin. Moreover, the difference in the magnitude of changes in postprandial glucose and insulin levels between the preload and the co-ingestion experiments implies that factors beyond the availability of circulating P, glucose and insulin may have been involved in improving of insulin sensitivity. The weak significant association seen between P preload on insulin sensitivity could be explained by the small sample size of the present study. On the other hand, glucose-phosphorus interaction in the proximal part of the small intestine may been involved in insulin sensitivity through incretin hormones. These hormones, especially glucagon like peptide-1 (GLP-1) and gastric inhibitory polypeptide (GIP) are known to be secreted in response to meal ingestion, especially high protein meals (rich in P) [9, 42] and were reported to affect insulin status and to play an important role in regulating postprandial blood glucose [9, 44, 45].

The observed improvement in the measures of insulin sensitivity following meal-phosphorus co-ingestion may have been partially involved in the reported synergic relationship between the intake of whole grains and glucose tolerance [46]. Especially since this relationship was not explained by the function of dietary fiber [47] and whole grains are rich in phosphors. Additionally, our observation may partially explain the observed parallel rise in metabolic syndrome with global urbanization and westernization of dietary habits, which favor low P intake [3]. In comparison to other studies in the literature, which have used P injections to study its effect on glycaemia and insulinemia [6], the current study used a different method that mimicked daily dietary habits, through the ingestion of 500 mg of P with a glucose load (approximately 1.7 mg of P per Kcal). Therefore, these findings highlight the role of P in improving the states of hyperglycemia and hyperinsulinemia in healthy individuals without the influence of serum calcium and FGF-23 which did not vary when P is used [25].

On the other hand, elevated fasting serum P levels were reported to be associated with mortality among patients with chronic kidney [48] and coronary [49] diseases. The association was partially explained by the capacity of high P conditions to induce vascular calcification and endothelial injury using in vitro studies [50–53]. Moreover, in a human (*in vivo*) study endothelial function impairment was apparent under high (1200 mg P/meal) and not normal (400 mg P/meal) P ingestion [54]. In fact, the negligible impact of dietary P intake on serum P levels implies that factors associated with increased serum P, rather than P intake, were probably behind the association between cardiovascular disease and serum P [24]. Recently, a weak association between dietary P intake and all-cause mortality was reported [55] and this was questioned since participants adopted different dietary patterns and P intake was not the only variable. Thus, the nature of the relation between P intake and cardiovascular disease and mortality is far from clear and requires further scrutiny [56].

In the present study, sample size was based on the previously reported data of AUC for glucose [57]. However, the observed large variations between subjects seem to have diluted the impact of the interventions. Further studies, using a larger sample size, would help in exploring the mechanisms by which the observed effects are mediated especially by examining the role of incretin hormones.

The limitation of this study lies in its small number of subjects enrolled into the experimental design. Moreover, although the dietary habits of these subjects were not assessed prior to the initiation of the study; however, as stated previously, fasting serum P status is not a good indicator of P intake. Postprandial status of the measured parameters is not likely to be affected by prior meal intake since all subjects were overnight fasted. Therefore, assessing dietary habits of subjects has no added implications on the study results.

## Conclusion

Ingestion of P with glucose was found to reduce postprandial glucose and insulin levels mainly at time 60 min. This was associated with a significant reduction in insulin sensitivity index. These changes are likely to have been attributed to phosphorus capacity to enhance insulin mediated peripheral phosphorylation, which is highly dependent on extracellular phosphorus availability or exogenous (dietary) phosphorus supply. Dietary phosphorus intake can exert its beneficial impact and prevent the deleterious effect of depleting intracellular phosphorus.

## Abbreviations

P: Phosphorus
G: Glucose
G + P: Ingestion of glucose and phosphorus
AUC: Area under the curve
